# Editorial: Executive functions in sports

**DOI:** 10.3389/fspor.2023.1340244

**Published:** 2023-11-29

**Authors:** Thomas Finkenzeller, Sabine Würth, Florian Heilmann, Franziska Lautenbach, Günter Amesberger

**Affiliations:** ^1^ Department of Sport and Exercise Science, University of Salzburg, Salzburg, Austria; ^2^ Faculty of Philosophy II, Institute of Sport Science, Martin Luther University of Halle-Wittenberg, Halle, Germany; ^3^ Institute of Sport Science, Humboldt-Universität zu Berlin, Berlin, Germany

**Keywords:** executive functions, strength, physical activity, expertise, cognitive training, athletes

**Editorial on the Research Topic** Executive functions in sports

## Introduction

The concept of executive functions has been an important focus for sport and exercise scientists for over two decades (1). During this period, substantial evidence-based knowledge has been accumulated regarding the interplay between exercise, sports, and cognitive processes. Nevertheless, several research questions still await comprehensive answers. A long-standing debate revolves around the development of exercise and training protocols that yield optimal short- and long-term effects on executive functions, especially for specific target groups, such as different age demographics and individuals with specific psychological conditions (2). A current area of interest is the examination of the relationship between sport-specific training, cognitive training, high sports expertise, and executive functions, which has recently led to great interest in sport-specific diagnostic tools for assessing executive functions (3, 4). The papers presented in this Research Topic aim to address aspects of those open research questions.

### Summary of selected articles from this research topic

Following a thorough review process, four original articles were accepted for publication. The 16 contributing authors represented diverse geographical backgrounds, including China, Spain, Italy, and Germany. Each article explores the theme of executive functions in sports from a different scientific as well as cultural perspective. This diversity is reflected in the applied research methodologies, cognitive assessments employed, and participant profiles, encompassing school pupils, college students, adolescents, and elite adult athletes. The two published cross-sectional and the two published intervention studies are summarized below.

### Cross-sectional studies

Zeng et al. examined the association between muscular strength and executive functions in children and adolescents in rural areas of China. In a cross-sectional study, 1,335 students aged 13–15 underwent a comprehensive battery of tests that included numerous strength measurements and executive function tasks. In summary, students with greater muscular strength exhibited shorter reaction times in executive functions and a reduced risk of developing executive dysfunction. The study results offer valuable recommendations for promoting the physical and mental well-being of children and adolescents.

The inhibition of initiated motor actions in response to new game situations is regarded as a crucial executive function for success in team sports. Fleddermann et al. investigated whether elite team sport athletes show improved motor inhibition with increased expertise. A sample of 106 elite athletes from various team sports completed the stop-signal reaction time task for hands and feet. The elite athletes with higher expertise demonstrated better motor inhibition than elite athletes of lower expertise. This effect was limited to motor inhibition of the hands. It is argued that the demands of the sport may have led to this result, as elite athletes of team sports were selected in which the use of the hands dominates.

### Intervention studies

Using a within-subject repeated measures design, Ballester-Ferrer et al. investigated the effect of acute exercise intensity on cognitive inhibition among female college students. This research examined the mechanisms underlying the cognitive response to physical exercise, considering both physiological and psychological parameters. Data analyses revealed that high-intensity interval training produced a significantly greater positive effect on inhibitory control compared to moderate continuous training and a non-exercise control condition. The improvement in inhibitory control was notably correlated with increased lactate release, indicating a connection between cognitive and physiological processes. This relationship was more pronounced in the congruent condition than in the incongruent condition of the Stroop task, suggesting that the enhanced cognitive function was primarily due to increased processing speed rather than improved cognitive inhibition. Additionally, the change in subjective vitality significantly correlated with the change in reaction time, but only in the congruent condition. These findings underscore the importance of considering physiological and psychological variables when investigating the exercise-cognition relationship.

Heilmann et al. concentrated on the application of cognitive training for executive functions in youth academy soccer players, addressing a common debate among researchers, applied sport psychologists, and coaches. This study investigated the effects of an eight-week smartphone-based, domain-generic cognitive training intervention on executive functions using a pre-post design with intervention and control groups. The results revealed that the evaluated smartphone game “Fruit Ninja” had no significant impact on working memory, inhibition, or cognitive flexibility. These findings align with the current body of research, which suggests inconclusive evidence regarding smartphone-based cognitive games' ability to enhance executive functions. The study also raises questions about the potential benefits of sport-specific cognitive perception tasks for improving executive functions, which, in turn, could enhance performance in sports-related situations.

### Future perspectives

The scientific works presented here provide significant contributions to our understanding of executive functions in sports while simultaneously prompting new research inquiries. Specifically, there is a call for in-depth exploration of the neurophysiological mechanisms underlying both acute and chronic effects to gain a clearer understanding of the specific impacts of exercise and sports on executive functions (5). This knowledge could prove useful in developing targeted exercise interventions aimed at enhancing specific sub-dimensions of executive functions. Additionally, it is recommended that emotional processes should be considered, as they play a substantial role in executive functions during mentally and physically demanding scenarios. Lastly, researchers should critically scrutinize the precise measurements taken when employing executive function tasks and how these measurements relate both practically and theoretically to the demands inherent in sports (6). It appears that there is a growing tendency within research to encompass the three core dimensions of executive functions, possibly stemming from an implicit standard within the research discipline.
